# The value of bronchial and cavity contraction rates in differentiating benign and malignant pulmonary cavities

**DOI:** 10.1186/s12890-020-01238-z

**Published:** 2020-08-06

**Authors:** Hua Zhang, Xia Qian, Zheng-Hua Liu, Yi Gong

**Affiliations:** Department of Radiology, the Third Affiliated Hospital of Nanchang University (also known as the First Hospital of Nanchang), No.128 Xiangshan north road, Donghu district, Nanchang, 330008 China

**Keywords:** Two-phase CT, Bronchial systole, Cavity shrinkage, ROC curve, Lung empty

## Abstract

**Background:**

The present study aimed to assess the value of bronchial and cavity contraction percentages in differentiating benign and malignant pulmonary cavities.

**Methods:**

Forty-two patients with pulmonary cavities were scanned by dual-phase computed tomography (CT). Then, the cavity and bronchial contraction percentages were respectively measured, the differences between the benign and malignant cavities were compared, and the best diagnostic critical point for differentiating benign and malignant cavities was obtained through the receiver operator characteristic (ROC) curve of the diagnostic test.

**Results:**

The contraction percentage of the bronchial end with benign cavities was significantly higher than that of the bronchial end with malignant cavities (*P* < 0.001). The contraction percentage was significantly higher in the benign group than in the malignant group (*P* < 0.001). The ROC analysis revealed that the sensitivity and specificity of the bronchial contraction percentage was 90.50 and 86.40%, respectively, while the sensitivity and specificity of the cavity contraction percentage was 90.50 and 90.90%, respectively.

**Conclusion:**

The dual-phase CT scanning of the bronchial and cavity contraction percentage can distinguish between benign and malignant cavities.

## Background

The pulmonary cavity can present with tissue necrosis, disintegration, and bronchial drainage, and mainly manifests as lung cancer, tuberculosis, and inflammatory cavities [1–3]. A benign cavity is comprised of granulation tissue, fibrous tissue, necrotic tissue, and various inflammatory cells. In addition to cancerous tissues, the trabeculae of damaged or residual bronchial cartilage and vascular connective trabecula can also be found in the wall of the cancerous cavity. Although there are obvious differences in pathology between these two diseases, there are many misdiagnoses (approximately 40%) due to the appearance of “same disease with different shadows, different disease with the same shadow” on the images. Since the treatment and prognosis of these two are quite different [4], determining how to accurately determine the nature of the disease is the key to clinical treatment. In order to improve the ability of imaging in differentiating benign and malignant cavities, the investigators compared the bronchial contraction percentage and cavity contraction percentage between benign and malignant cavities.

## Methods

### Subjects

From January 2014 to December 2015, 42 patients with pulmonary cavities, who were treated in our hospital, were enrolled in the present study. Among these patients, 20 patients had benign cavities, and 22 patients had malignant cavities. The ages of these patients ranged from 25 to 79 years, with an average age of 60.25 ± 12.92 years. All patients were confirmed by pathology, and some benign patients were diagnosed with a benign cavity after clinical treatment. The present study was approved by the Ethics Committee of our hospital, and all patients provided signed informed consent.

### Inclusion and exclusion criteria

Inclusion criteria: (1) patients with a definitive diagnosed pulmonary cavity, and (2) patients > 18 years. Exclusion criteria: (1) patients with severe liver and kidney dysfunction, and (2) lactating or pregnant patients.

### Inspection methods

A Philips BRILLIANCE 64-slice spiral computed tomography (CT) machine and data post-processing workstations were used for the present study. The data acquisition channel was 64 slices × 0.625 mm, the frame rotation cycle was 0.4–0.5 s, the tube voltage was 120–135 kV, and the tube current was 400 mA. Before scanning, patients were trained to perform breath-hold scanning after inhalation and exhalation, respectively, and these patients were informed of the examination process in detail. Patients underwent the scanning in the supine position, and both arms were held up and covered the head, which entered the scanning space first. The scanning ranged from the apex of the lung to the lower boundary of the lung. The breath-holding non-enhanced scans were performed in the inspiratory phase and expiratory phase, respectively. The scanning time was approximately 12 s. The multiplanar or surface reconstruction of the post-processing workstation was used to display the bronchi connected with the cavities, and the diameter of the bronchial end with the cavities, the left-right inner diameters of the cavity, and the front-back inner diameter of the cavity were measured, respectively.

### Image analysis

Three physicians, each with more than 10 years of clinical experience, measured the bronchial diameter and the left-right and front-back diameters of the cavity on the images of each patient acquired by the inspiratory and expiratory phase scanning, and the data were averaged. The bronchial contraction percentage = inner diameter of the bronchial end with cavities in the inspiratory phase/inner diameter of the bronchial end with cavities in the expiratory phase. The cavity contraction percentage = the inspiratory/expiratory phase (the maximum left-right inner diameter of the cavity × the maximum front-back inner diameter of the cavity). The data were recorded, and radiologists read the films to differentiate the benign and malignant nature of the lesions. These results were then compared with the pathological and clinical results.

### Statistical methods

Data were analyzed using statistical software SPSS 17.0. Measurement data were expressed as mean ± standard deviation (x ± SD), and count data were expressed as a percentage (%). The variables were compared between the two groups using a *t*-test. Non-normally distributed means or normally distributed means with the heterogeneity of multiple samples were evaluated using the non-parametric test. Count data were compared using an *X*^*2*^-test. The sensitivity, specificity, and threshold of the contraction percentage of the bronchial diameter, and contraction percentage of the bronchial end with the cavity were analyzed using the receiver operator characteristic (ROC) curve. *P* < 0.05 was considered statistically significant.

## Results

### General information

In the present study, 42 eligible patients were selected from the 200 patients with pulmonary cavities. Among these patients, 22 patients had malignant cavities, and 20 patients had benign cavities. The age of these patients ranged from 25 to 79 years, with an average age of 60.25 ± 12.92 years.

### Comparison of contraction percentage of the diameter of the bronchial ends with benign and malignant cavities

**Fig. 1:**
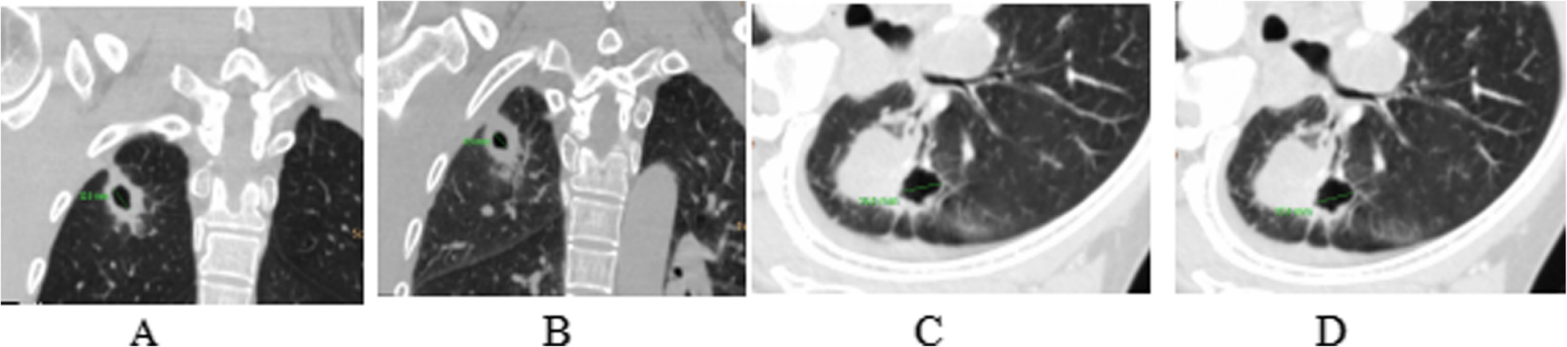
Typical CT findings. **a** and **b** were the same patient, male, 62 years old, with recurrent cough and sputum for more than 1 year, which was confirmed to be pulmonary tuberculosis by pathology. **a**: The maximum diameter of the cavity after deep inspiration was 12.0 mm. **b**: The maximum diameter of the cavity after deep exhalation was 8.9 mm. **c** and **d** were another patient, male, 67 years old, cough for more than 2 months, confirmed by pathology as lung cancer. **c**: The maximum diameter of the cavity after deep inspiration was 16.0 mm. **d**: The maximum diameter of the cavity after deep exhalation was 15.9 mm

**Fig. 2:**
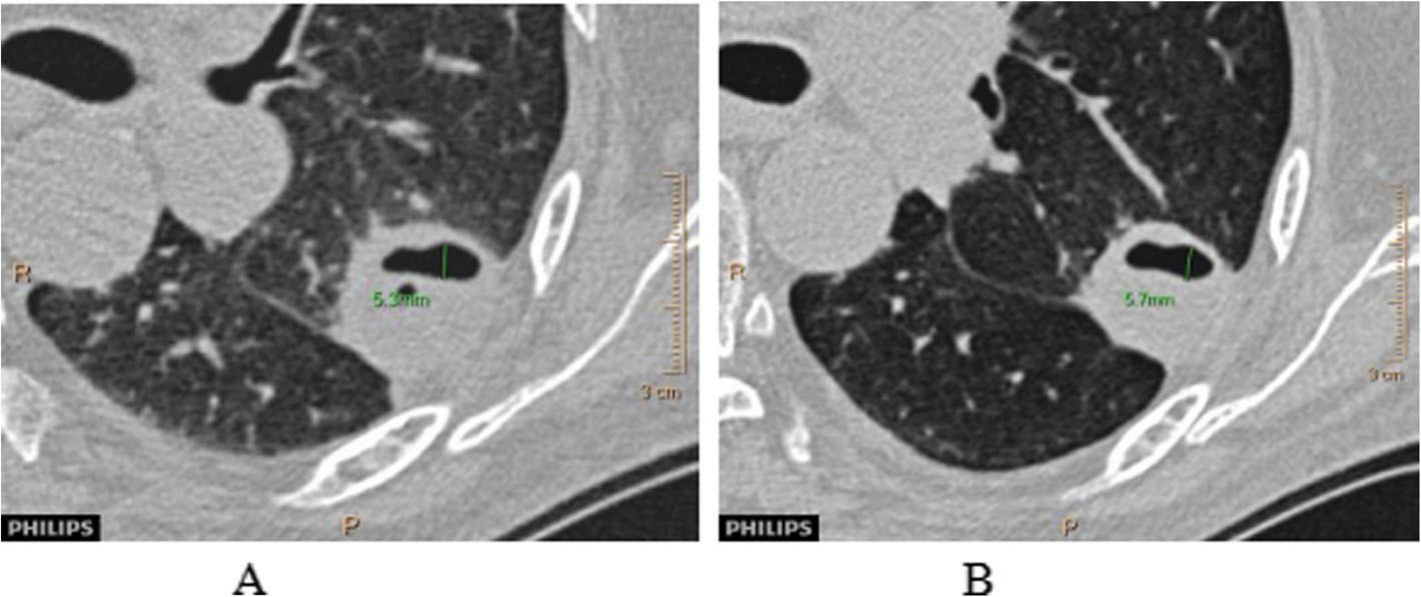
The changes in lung volume. **a**: The scanning image of breath-holding after deep exhalation. **b**: Scan image of holding breath after deep inhalation

**Fig. 3:**
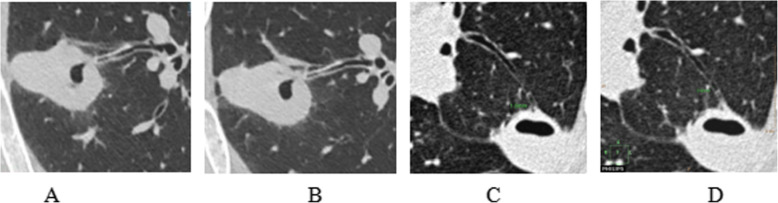
Inner diameter of the bronchial tube. **a** and **b** were the same patient, male, 72 years old, hemoptysis for more than 1 week. Lung cancer was confirmed by pathology. **a**: The maximum inner diameter of the bronchial tube at the hollow end of the inspiratory phase was 1.20 mm. **b**: The maximum inner diameter of the bronchial tube at the hollow end of the expiratory phase was 1.18 mm. **c** and **d** were the same patient, female, 58 years old, coughing for more than 1 month. Tuberculosis was confirmed by pathology. **c**: The maximum diameter of bronchial tube at the hollow end of inspiratory phase was 1.00 mm. **d**: The maximum diameter of bronchial tube at the hollow end of expiratory phase was 0.90 mm

**Fig. 4:**
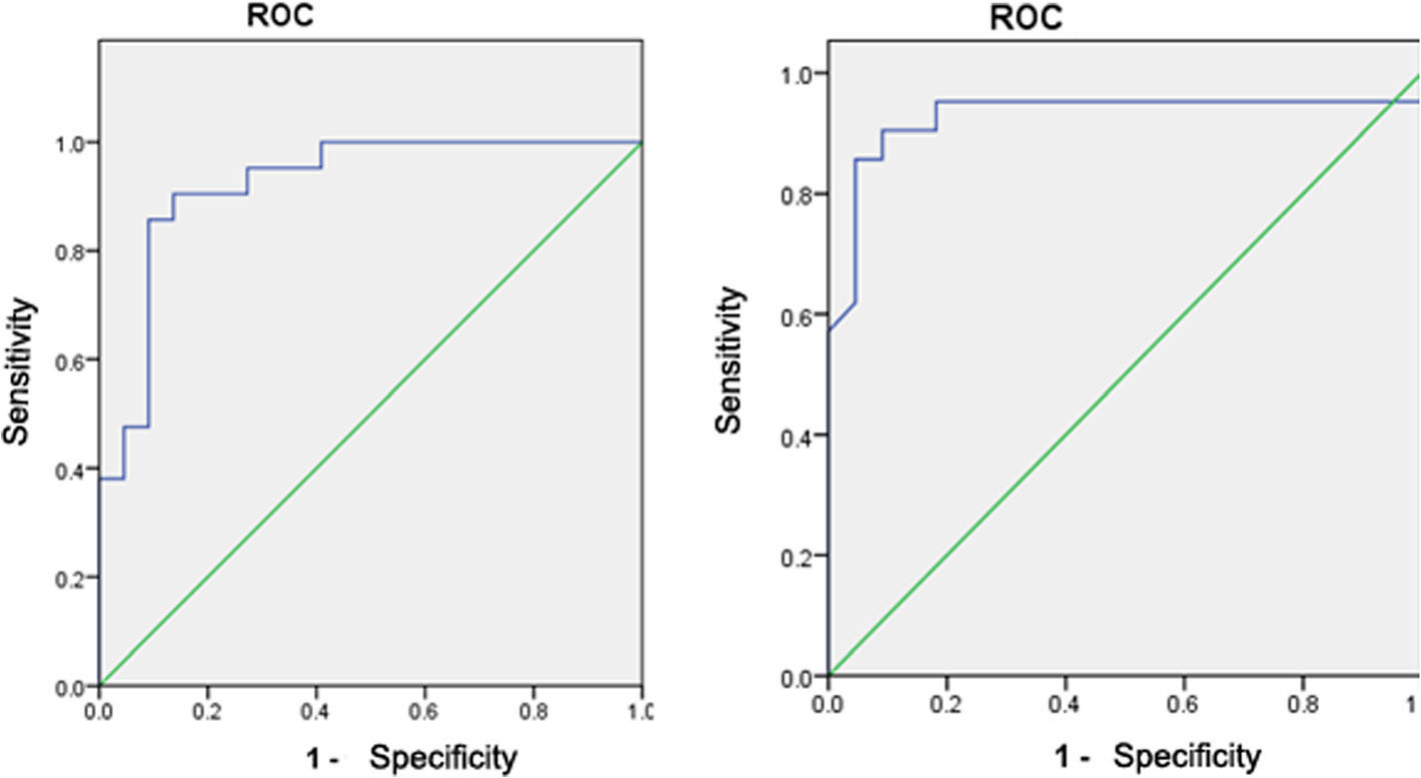
The ROC curves of the bronchial contraction percentage and cavity contraction percentage for predicting benign and malignant cavities

The contraction percentage of the bronchial end with benign cavities was 1.504 ± 0.349 in 20 patients with benign cavities, while the contraction percentage of the bronchial end with malignant cavities was 1.082 ± 0.164 in 22 patients with malignant cavities. The difference in contraction percentages between benign and malignant cavities was statistically significant (t’ = 4.935, *P* < 0.001; Table 1). The cavity contraction percentage was 1.29 ± 0.25 in the benign group and 1.02 ± 0.06 in the malignant group. The differences were statistically significant (t’ = 4.603, *P* < 0.001). The ROC curve for the diagnostic test revealed that the area under the curve (AUC) of the bronchial contraction percentage was 0.922 (95% confidence interval). When the best diagnostic threshold of the bronchial contraction percentage was 1.111, the sensitivity was 90.50%, and the specificity was 86.40%. The AUC of the cavity contraction percentage was 0.927 (95% confidence interval). When the best diagnostic threshold of the contraction percentage of the cavity was 1.08048, the sensitivity was 90.50%, and the specificity was 90.90% (Fig. 1, 2, 3). The ROC curves of the bronchial contraction percentage and cavity contraction percentage for predicting benign and malignant cavities are presented in Fig. 4.

**Table 1:** ROC Curve Analysis of Cavity contraction percentage

Item	Youden Index	AUC	Sensitivity	Specificity	Best diagnostic threshold
Bronchial contraction percentage	0.769	0.922	0.905	0.864	1.111
Cavity contraction percentage	0.814	0.927	0.905	0.909	1.08048

### Pathological and clinical results

Among the 42 patients with cavities, 20 patients had benign cavities. Among these patients, 15 patients had tuberculosis, five patients had an abscess, and one patient had tuberculosis and fungal infection. The remaining 22 patients had malignant cavities. Among these patients, 14 patients had adenocarcinoma, seven patients had squamous cell carcinoma, and one patient had large cell lung cancer.

## Discussion

The results of the present study revealed that the contraction percentage of the bronchial end with benign cavities was significantly higher than that of the bronchial end with malignant cavities (*P* < 0.001). Furthermore, the contraction percentage was significantly higher in the benign group than in the malignant group. The ROC analysis revealed that the sensitivity and specificity of the bronchial contraction percentage were 90.50 and 86.40%, respectively, and the sensitivity and specificity of the cavity contraction percentage were 90.50 and 90.90%, respectively.

Typical benign and malignant pulmonary cavities are easy to diagnose due to the differences in the shape, density, and surrounding conditions of the lesions on the images. However, in practice, less than 60% of these cavities can be clearly identified as benign or malignant. Therefore, some scholars adopted CT-guided thoracic vertebra puncture to make a clear diagnosis [5, 6]. However, CT-guided thoracic puncture may lead to tissue damage [7, 8]. Besides, the histopathological features of the pulmonary cavity were varied, and the clinical prognosis of the pulmonary cavity resulting from different causes were also different. Therefore, it is important to differentiate between benign and malignant pulmonary cavities. Furthermore, it is valuable to explore new safe and effective methods to differentiate benign and malignant cavities.

A cavity forms due to the discharge of necrotic tissue caused by various reasons from the drainage bronchus, and its formation mechanism is complicated. These can include the degenerative necrosis of tumor cells, the physiological cell death mechanism of squamous cell carcinoma, drugs or radiotherapy, and the autolysis of tumor cells due to the enzymatic system [9]. Previous studies have revealed that the maintenance of intrapulmonary bronchial diameters depends on the balance between the contraction of elastic fibers and smooth muscles of the bronchial wall and the tension of surrounding lung tissues [10–12]. An experiment verified that with repeated branching of the bronchi, the number of cartilage rings decreased, the number of smooth muscles increased, and the influence of intrathoracic pressure on the bronchial diameter increased. Bronchial smooth muscles affect airway contraction. Both benign and malignant pulmonary cavities form due to the excretion of necrotic material from necrotic tissues [13–15]. The bronchial wall of the malignant cavity thickens and becomes stiff due to tumor infiltration. When adding the effect of the traction of the fibrogenic reaction in tumors, the bronchus within the tumor is not only not flattened by the tumor, but also maintains a high degree of patency, and even expands. The benign cavity would continue to have a very soft wall since it is not affected by tumor invasion and fibrosis reaction. Therefore, the degree and manner of destruction of these two on the bronchioles differ, resulting in varying degrees of enlargement and reduction of bronchioles. The contraction percentage of the bronchial ends with benign and malignant cavities would differ to a certain extent.

Since the malignant cavity wall was mainly composed of the residual necrotic tumor tissue, and the surface was rough, the cavity elasticity was poor. Furthermore, since the wall of the malignant cavity is relatively thick, and most of the walls of the cavity are close to the edge of the lesion, the growth of tumor cells is relatively active, the density of tumor cells is high, and the stroma of tumors is relatively abundant. Moreover, these also lead to weak elastic contractions of the cavity wall. There are many kinds of benign cavities. Most of these are caused by chronic pulmonary infections, such as tuberculosis and suppurative and fungal infections. The cavity wall of benign cavities is composed of granulation tissue, fibrous tissue, necrotic tissue, and differing proportions of various inflammatory cells, and the cell density is similar to that of normal tissue. Therefore, the degree of elastic contraction of the cavity is close to that of the normal tissue. Furthermore, most of these cavities are relatively smooth and thin and have a relatively strong centrifugal elastic contraction. Therefore, the centrifugal elastic contraction of these cavities was relatively strong, and the differences in elastic contraction between benign and malignant cavities were statistically significant. With the rapid development of multi-slice spiral CT, ultra-high quality, and clear thin-slice isotropic CT images can be obtained, and fine image features of the lung can be displayed [16]. This can be used to measure the internal diameter of bronchioles and cavities accurately. Therefore, it is convenient for observing the correlation between the cavity and its surrounding bronchioles [2, 17–19].

In the present study, breath-hold scanning after inhalation was only used to diagnose. In the diagnoses of 42 patients with cavities, the correct result was obtained in 22 patients. Furthermore, dual-phase scanning was also used to diagnose. When the best diagnostic threshold of the bronchial contraction percentage was 1.111, the sensitivity was 90.50%, and the specificity was 86.40%. When the best diagnostic threshold of the contraction percentage of the cavity was 1.08048, the sensitivity was 90.50%, and the specificity was 90.90%. According to the contraction percentage of the diameter of the bronchial end with cavities, large cavities were diagnosed as benign cavities, while small or unchanged cavities were diagnosed as malignant cavities. In the diagnosis of 42 patients with cavities, the correct result was obtained in 41 patients, while one patient with lung cancer was misdiagnosed as having a tuberculous cavity. After analyzing the misdiagnosis, it was found that the reason may be because the patient coughed and expectorated many times during the scan, and some of the cavity contents were discharged into the bronchus or out of the body, resulting in the direct increase in the inner diameter of the cavity. However, this was not correlated to the contraction of the cavity itself. The results of the present study revealed that the bronchial and cavity contraction percentage was significantly higher in the benign group than in the malignant group. This suggests that bronchial and cavity contraction percentages are helpful for judging the benign and malignant nature of the cavity. This examination has the advantages of simple, non-invasive operations, at low cost, with fast diagnosis and high accuracy. Hence, it is worthy of vigorous wider clinical use and application.

The benign cavities were mainly caused by tuberculosis and abscesses, while the malignant cavities were mainly caused by adenocarcinoma and squamous cell carcinoma. The pathology of lung cancer with cavitation was caused by the lack of blood supply in the central lesion of the tumor tissue and the occurrence of ischemia and necrosis, resulting in the formation of a cavity. The wall of the cavity was mainly composed of the tumor tissue with necrosis and residue, which was mostly biased. Therefore, CT showed an uneven thickness of the wall of the cavity, with the inner wall uneven or nodular (see Fig. 1). The benign cavities were mainly caused by tuberculosis and abscesses, which were caused by liquefaction of the lesion, discharge of necrotic material by communicating bronchus, and air entering the cavity. A small amount of caseous necrotic tissue or liquid can be seen in the cavity, and the inner wall was mostly smooth. Therefore, the CT appearance of the cavity was a thick wall with a smooth inner wall, and sometimes gas-liquid or liquid-liquid planes can be seen in the cavity. Since the wall of the cavity was composed of granulation tissue, a CT-enhanced scan showed obvious annular enhancement of the wall.

The present study provides a strong basis for differentiating benign and malignant pulmonary cavities. However, it also has some limitations. First, the present study is not a randomized controlled trial, and there was still a certain risk of bias. Second, the present study is a single-center clinical trial that included a small sample size. Hence, further large sample sizes and multi-center clinical trials are needed. Third, the patients had poor breathing coordination, showing that the diagnostic results had obvious errors. Finally, the bronchial and cavity contraction percentages were affected in patients with underlying lesions. In the present study, some corresponding measures were also taken to minimize the influencing factors, including breathing training before scanning. Furthermore, for patients with underlying diseases, a comprehensive evaluation method was adopted. A total of 20 patients with benign cavities and 22 patients with malignant cavities were included in this study. The study did not include a control group. Another trial with a control group is needed in the future. Besides, the time of 12 s referred to the average of about 12 s of patients holding their breath during the scan. Patients with poor breath-holding were not suitable for this test, which is a disadvantage of this study. The present research team will further improve this aspect of the present study.

## Conclusion

Dual-phase CT scanning of the bronchial cavity and cavity contraction percentages can distinguish between benign and malignant cavities. This examination has the advantages of simple, non-invasive operations, low costs, fast diagnoses, and high accuracy. This is worthy of a wider clinical application.

## Data Availability

We declared that materials described in the manuscript, including all relevant raw data, will be freely available to any scientist wishing to use them for non-commercial purposes, without breaching participant confidentiality. The data used/generated in our study is available from the corresponding author.

## Abbreviations

CT: Computed tomography
ROC: Receiver operator characteristic
AUC: Area under the curve
